# Blended Learning: The impact of blackboard formative assessment on the final marks and students’ perception of its effectiveness

**DOI:** 10.12669/pjms.36.3.1925

**Published:** 2020

**Authors:** Mukhtiar Baig, Zohair Jamil Gazzaz, Mohammed Farouq

**Affiliations:** 1 Dr. Mukhtiar Baig, Ph.D. Department of Clinical Biochemistry and Assessment Unit, Faculty of Medicine, Rabigh, King Abdulaziz University, Jeddah, Saudi Arabia; 2 Dr. Zohair Jamil Gazzaz, Ph.D. Department of Medicine, Faculty of Medicine, Rabigh, King Abdulaziz University, Jeddah, Saudi Arabia; 3 Dr. Mohammed Farouq, ABP. Department of Pediatrics, Faculty of Medicine, Jeddah, King Abdulaziz University, Jeddah, Saudi Arabia

**Keywords:** Blackboard, Blended learning, Medical students, Formative assessment, Students’, perception

## Abstract

**Objective:**

To explore the impact of Blackboard (Bb) formative assessment on the final score in the endocrine module and determine the medical students’ perception of the impact and effectiveness of Bb.

**Methods:**

This exploratory case study was carried out at the King Abdulaziz University (KAU), Jeddah, Saudi Arabia (SA). Blackboard was used in the course management and formative assessment of third-year medical students and three years of data was collected (2016, 2017, 2019). In the last week of the module before the final exam, a formative assessment test that comprised of 50 Multiple Choice Questions (MCQs) was posted on Bb each year. All the students filled a questionnaire regarding their perception about the impact and effectiveness of Bb.

**Results:**

Overall, summative exam scores were significantly higher than the scores in formative assessment (p <0.001). A substantial positive correlation was observed between students’ marks in the online (Bb) MCQ exam and their final exam marks (p <0.001). Regarding the features of Bb, most often used by the students’ were course resources uploaded on the Bb, assignments, online quizzes, and others. Majority of the students were satisfied with the use of Bb in this module.

**Conclusions:**

The majority of the students liked this blended learning (BL) method and conceded the impact and effectiveness of Bb. The formative online assessment on Bb improved the students’ performance in the final exam and a positive correlation was noted between students’ marks in online (Bb) exams with their final exam marks.

## INTRODUCTION

Usually, medical education in the Saudi Arabia (SA) is face to face, and the use of blended learning is not very common among medical students. However, all the public sector universities have access to Blackboard and assessment software “question mark.” Use of learning management system (LMS) in education is gradually becoming popular among the faculty members and students in SA. Nevertheless, the faculty at medical colleges is not adequately taking advantage of the LMS. This could be due to the peculiar nature of medical training, which requires face-to-face training, hospital ward rounds, and interaction with the patients. The use of electronic gadgets and digital media are widespread among Saudi students. Recently, a study from King Abdulaziz University (KAU) reported that students like medical websites, online textbooks, and journals.^1^ The KAU students use online medical applications for their knowledge acquisition and to do their assignments^2^, and a significant number of medical students are addicted to their smartphones.^3^

Blended learning (BL) is the integration of conventional face-to-face teaching with online content.^4^ It is extensively implemented across higher education, with several academicians mentioning it as the “new traditional model” or the “new normal” in course delivery. 5 LMS is being used worldwide in universities for teaching and learning activities; thus, information technology plays an important role in learning procedures. 6 The Bb is a specially designed e-learning platform and course management system, and it is an important model of the virtual learning environment (VLE).^7^ The Bb has several advantages. It provides online assessments, course organization and distribution, assignment administration, student tracking, and virtual collaboration, all of which augment a teaching and learning environment. A study from King Saud University, SA, reported that the learning features of Bb were not used efficiently by medical students, and they reported technical difficulties while utilizing it.^8^ Literature indicates that the use of interactive technological strategies enriches the students’ learning.^9^

The use of Bb is easy and playful because the course material is available anywhere and anytime.^8^,^10^ The objective of this study was to explore the medical students’ perception of the impact and effectiveness of Bb and determine the effect of Bb formative assessment on the final score in the endocrine module.

## METHODS

The current exploratory case study was carried out at the Faculty of Medicine, Rabigh, KAU, Jeddah, SA. (FMR-04-37-H, Dated: 15-12-2015) The Research Ethics Committee of Faculty of Medicine, Rabigh, KAU, Jeddah, approved the research and data were collected after obtaining informed consent from all the students. The KAU uses the Bb as a Learning Management System (LMS). In the endocrine module, Bb was used in the course management and formative assessment of third-year medical students and three years of data was collected (2016, 2017, 2019). The data was not collected in 2018 because of some technical problems.

Our medical college is relatively a newly established college and is located at the coastal area of the Red Sea in a small town of the Western region of the KSA.^11^ There were 36, 38, 38 male students enrolled in 2016, 2017, and 2019, respectively, at the Faculty of Medicine, Rabigh. Several popular options of the BB such as content collection, discussion board, instant feedback, announcement, emails, grade book, course calendar, and tests and quizzes were used for the third-year medical students. The study guide, timetable, lecture PowerPoint slides, reference materials, and several questions in the discussion board were posted during the endocrine module. In the last week of the module before the final exam, a formative assessment test comprised of 50 MCQs was posted on Bb each year and after the final exam; the impact of formative assessment was determined on the final marks in the module exam. After finishing module activity, each year, all students were invited to fill a structured questionnaire and almost all students’ returned a completed questionnaire. The questions regarding the impact and effectiveness of Bb for this study were taken from an already published study after the authors’ approval.^12^

### Statistical Analysis:

Data was analyzed on SPSS version 23. Categorical variables were calculated as frequency and percentage while numerical variables as mean±SD. Students’ t-test was used to compare the scores of formative assessment and final exam scores, and Pearson’s correlation was used to find the correlation. A two-sided p-value <0.05 was considered significant. Open-ended answers were analyzed for repetitive sequences by the researchers.

## RESULTS

**Fig.1:**
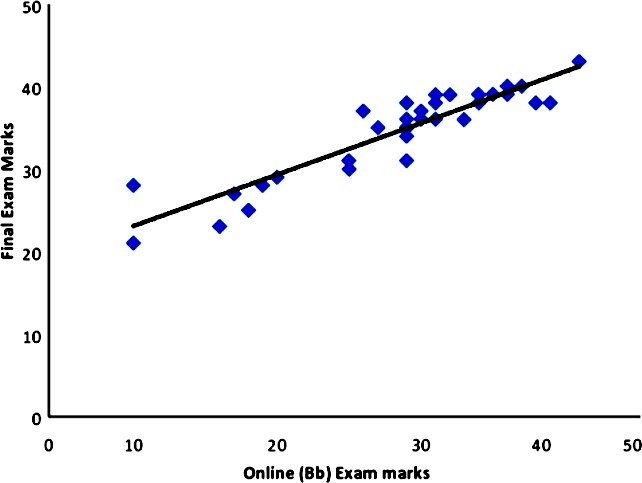
Correlation between final exam marks and online (Bb) exam marks.

Overall, the exam score was significantly higher in all three years relative to the formative assessment (p <0.001) (Table-I). A positive correlation was found between students’ performance in online (Bb) MCQ exam and their final MCQ exam (p <0.001) (Fig.1).

**Table-I:** Difference in students’ scores in formative and final exam.

Year	Score in formative assessment Total marks=40	Scores in final exam Total marks=40	P-value
2016	28.89±7.79	34.86±5.31	<0.001
2017	30.31±5.25	36.31±3.91	<0.001
2019	29.67±4.25	34.47±3.23	<0.001

Regarding the features of Bb, most often used by the teachers and students’ are shown in Table-II. Students responses regarding use of Bb in the endocrine module are shown in Table-III.

**Table-II:** Students responses regarding features of Bb the instructor and students used during the endocrine module.

Statements	Always N(%)	Sometime N(%)	Never N(%)
*During course instruction, what features of Bb your instructors regularly used:*			
posting syllabi on Bb	90(90)	10(10)	0
assignment or assessment feature	75(75)	25 (25)	0
online tests	30(30)	51(51)	19(19)
for reporting grades using the grade book feature	48(48)	35(35)	17(17)
for communicating by discussion tab or the chat rooms	100(100)	0	0
*Which tools of communication the instructors used in Bb:*			
Mail	60(60)	30(30)	10(10)
Announcements	75(75)	20(20)	05(05)
Chat	29(29)	21(21)	50(50)
Discussions	72(72)	20(20)	08(08)
Calendar	10(10)	17(17)	73(73)
*Instructors used the assorted features and tools on Bb effectively:*			
Syllabus	73(73)	12(12)	15(15)
resources/web links	77(77)	17(17)	06(06)
mails/messages	25(25)	20(20)	55(55)
my grades	45(45)	17(17)	38(38)
assignments	82(82)	14(14)	04(04)
*During course instruction, what features of Bb you regularly used:*			
Resources such as e-books, ppt, reading material, websites	82(82)	18(18)	0
Communicate with classmates	10(10)	25(25)	65(65)
Turn in assignments	50(50)	34(34)	16(16)
Take online quiz/s	80(80)	18(18)	2(2)
To check grades	51(51)	34(34)	15(15)

**Table III:** Students perceptions regarding use of Bb in the endocrine module.

Statements	Strongly Agree N(%)	Agree N(%)	Neutral N(%)	Disagree N(%)	Strongly Disagree N(%)
Computers based assessment is easier than paper pencil test	30(30)	45(45)	08(08)	12(12)	05(05)
E-assessment enhanced my learning	35(35)	32(32)	12(12)	13(13)	08(08)
I have no problem accessing Bb from home.	40(40)	25(25)	10(10)	15(15)	10(10)
I check Bb at least once a day.	32(32)	29(29)	20(20)	08(08)	11(11)
I like the idea of having online exams, quizzes, class activities, etc.	56(56)	19(19)	11(11)	09(09)	05(05)
Using online learning tool has improved my technical skills	32(32)	19(19)	20(20)	09(09)	20(20)
I feel more comfortable posting my opinions on the discussion board rather than to speak up in class.	29(29)	32(32)	10(10)	15(15)	14(14)
Using online learning make me less dependent on my teachers for help.	24(24)	26(26)	12(12)	17(17)	21(21)
I become more confident in expressing my ideas using communication technologies such as email, chat, and discussion forum.	37(37)	38(38)	08(08)	11(11)	06(06)
I am satisfied with the use of Bb in this module	65(65)	09(09)	16(16)	05(05)	04(04)

To the open-ended questions on “any suggestion/s or recommendations for helping instructors to improve their use of Bb for course instruction,” 52% recommended using it in all courses and 52% recommended its use for more formative assessment. When asked about “What you liked most about using Bb**”,** 71% suggested easy of use. Of particular note, 29% of students disliked using Bb because of the deadline of the assignments and the presence of numerous tabs (18%) and the requirement to training (22%). Sixty-seven percent of students were satisfied with their experience of using Bb (not shown in table).

## DISCUSSION

We found that the final exam score in the endocrine module was higher as compared to the online quiz as formative assessment in the endocrine module. A study reported that more engagement of students with online materials improves the students’ test scores; it also reported a robust relationship between discussion board activity and final marks.^13^ A German study reported that, among medical students, the purpose of using Bb was to organize study info, exam preparation, and planning and post-processing of lessons.^14^ In contrast to our results, the use of Bb for communicating with other students or teachers or keeping lists or calendars is very low; moreover, their overall daily use was 38.6%, weekly use was 48.3%, and 13.1% used it less than once a week.^14^

Our study found a positive correlation between students’ marks in online (Bb) MCQ exam and their final MCQ exam marks. These results concur in several previous studies.^15^,^16^ In contrast to our study, another study reported that online quizzes on Bb interface in anatomy and physiology subjects did not consistently improve students’ performance in comprehensive examinations.^17^ Our finding is comprehensible because the online MCQ exam was held a few days before the summative exam, so the students improved their weaknesses and removed their misconceptions and thus obtained better results. Two previous studies found that the higher use of course management system (CMS) usage was associated with better student exam performance^18^,^19^ while another study found no correlation between usage and final marks.^16^ The use of e-learning encourages the student to contact the material several times at his/her own pace and convenience till they master the content, and because of the “permanency of discussions,” students can consult the material at any time.^20^ Additionally, BL not only surpasses the constraints of site and time but also assists instruction approaches that are difficult to accomplish with textbooks.^21^ The learning flexibility provided by the online part of BL gives it superiority over only traditional teaching.

The present study reports that user activity on Bb was more on course content folder as well as assignments and quizzes. These results are comparable to a study by Griffiths and Graham.^22^ Contradictory to our results, a study pointed out that majority of student activities within the LMS were linked with the management of document and communications and a very small number of students (5%) were involved in the more collaborating parts of the LMS like use of discussion board, wikis, or chat.^23^

There are several tools to measure the effectiveness of the course delivery, and students’ satisfaction is one of them. There are many empirical pieces of evidence found in the literature that describe the positive attitudes of healthcare students toward e-learning.^16^ An Australian study reported that medical students extensively used digital self- directed learning resources, including Bb and all students attempted formative assessment on Bb; they stated that e-learning resources were beneficial.^24^ Turkish study results indicated that medical students perceive the BL environment positively.^25^ Another study suggested that the majority of the students liked BL because in this way inadequacy in one method can be compensated by the other.^26^ The use of only one method makes the teaching monotonous, and students lose interest and concentration in a few minutes while the combination of different teaching and learning methods improves the engagement with the content, comprehension, and retention of knowledge.^27^

The present study results regarding the impact and effectiveness of Bb are similar to other studies.^12^,^28^ A study suggested that the use of LMS have less effect on cooperative and communicative learning.^23^ Our study results indicate that students liked the use of LMS in the endocrine module and recommended its use in other modules as well. Medical education is already a content-loaded field, and faculty and students are always busy in several module activities simultaneously. So the web-based activities and module contents, assignments and quizzes on Bb would make their life less stressed. They enjoyed the opportunity provided for involvement in the course activity anytime and anywhere. Moreover, they felt confident in expressing their ideas using the discussion board.

A study pointed out that, although students liked the use of web-based learning besides the conventional style, they were generally frightened of its potential as an alternative to face-to-face teaching.^16^ They elaborated that, in the past years of acquiring knowledge in traditional methods and backgrounds, the supposed necessity of face-to-face learning may have been deep-seated in their minds. Therefore, shifting from conventional approaches to the online method would compel them out of their comfort zone, making them feel stressed and anxious. Therefore, the change should be gradual and cautiously planned, and instead of using only online courses it is far better to use BL courses.

A recent Pakistani study pointed out several issues faced during blended learning and proposed that encouragement, persistent support, appropriate feedback and convenient accessibility of the tutors can help students to overcome all the challenges faced by the students during shifting towards BL.^29^

### Limitations of the Study

Our study is limited to a single module and it did not investigate the interest of the student and time given to their study. So these factors limit the generalizability of our results.

## CONCLUSION

The majority of the students liked the BL method and conceded Bb’s impact and effectiveness. The formative online assessment on Bb improved the students’ performance in the final exam, and a positive correlation was noted between students’ marks in online (Bb) exam with their final exam marks.

### Authors Contribution:

**MB:** Conceived the idea, designed research, analyzed data, drafted manuscript and responsible and accountable for the accuracy and integrity of the work.

**ZJG, MF:** Contributed in research design, edited and revised manuscript.
